# Unicameral Bone Cyst: Diagnosis, Treatment, and Follow-Up Methods, a Multicenter Study

**DOI:** 10.3390/medicina62071320

**Published:** 2026-07-08

**Authors:** Ali Erkan Yenigül, Mahmut Kürşat Özşahin, Osman Emre Aycan, Bahattin Kerem Aydın, Evrim Şirin, Şahin Çepni, Ahmet Nadir Aydemir, Mehmet Bartu Sarısözen, Bülent Erol

**Affiliations:** 1 Department of Orthopedics and Traumatology, Bursa Uludağ University, 16059 Bursa, Turkey; bartu@uludag.edu.tr; 2 Department of Orthopedics and Traumatology, Istanbul Cerrahpasa University, 34320 Istanbul, Turkey; drmkursatozsahin@yahoo.com; 3 Department of Orthopedics and Traumatology, Metin Sabancı Baltalimanı Bone Diseases Training and Research Hospital, 34470 Istanbul, Turkey; emre_md@yahoo.com; 4 Department of Orthopedics and Traumatology, Konya Selçuk University, 42075 Konya, Turkey; bkaydin@yahoo.com; 5 Department of Orthopedics and Traumatology, Marmara University, 34034 Istanbul, Turkey; evrim.sirin@marmara.edu.tr (E.Ş.); bulerol@hotmail.com (B.E.); 6 Department of Orthopedics and Traumatology, Ankara Bilkent City Hospital, 06800 Ankara, Turkey; ortdrsahin@yahoo.com; 7 Department of Orthopedics and Traumatology, Pamukkale University, 20070 Denizli, Turkey; anaydemir@yahoo.com

**Keywords:** unicameral bone cyst, multicenter, treatment, percutaneous injection

## Abstract

*Background and Objectives*: Unicameral bone cyst (UBC) is a benign cystic lesion originating from the metaphysis, often extending toward the diaphysis as the individual grows. It is more common in boys aged 5–15 years, typically affecting long bones like the humerus and femur. UBC is usually asymptomatic and found incidentally on imaging, with pathological fractures being a common complication. Treatment options include conservative observation, percutaneous injection, and surgery. This study aims to compare the diagnostic, treatment, and follow-up processes of UBC cases across multiple centers in our country. *Materials and Methods*: This study reviewed 180 UBC cases in patients aged 0–20 from seven clinical centers in our country. Patient demographics, symptoms, diagnostic methods, treatment approaches, and follow-up data were collected. Radiological and histopathological diagnostic accuracy, treatment methods (conservative, percutaneous injection, surgical), and recovery were analyzed using Musculoskeletal Tumor Society (MSTS) scores and Capanna criteria. *Results*: Among 180 patients, 50% had pain. Most diagnoses were made through radiographic imaging, with 96.1% diagnostic accuracy. The humerus (44.7%) and femur (30.7%) were the most commonly affected bones. Pathological fractures were found in 35% of cases. Treatment included conservative (19%), percutaneous injection (31.8%), and surgery (49.2%). The average follow-up period was 81 months, with early complications being skin issues and late complications primarily limb shortening. *Conclusions*: The study highlights the effectiveness of different treatment approaches for UBC, showing that surgical treatment offers the best clinical outcomes, while percutaneous injection is a less invasive option. Treatment should be tailored to individual patient needs.

## 1. Introduction

A unicameral bone cyst (UBC), also known as a “simple” or “solitary” bone cyst, is a cystic lesion that originates in the metaphysis and expands towards the diaphysis as the individual grows. These benign lesions manifest as lytic features on radiological examination and may present as unicameral or multilocular structures, accompanied by septations [1,2,3,4]. It typically affects boys aged 5–15 (M:F ratio 2:1) and can be observed in all bones, with the most common location being the proximal humerus (50%) [1]. UBC is predominantly a disease of childhood and adolescence, with the highest incidence reported during the first two decades of life. Although UBCs are benign and non-neoplastic lesions, their clinical relevance is related to progressive bone expansion, cortical thinning, and the risk of pathological fracture. The exact etiology remains unclear. The most widely accepted hypothesis suggests that impaired venous drainage in the metaphyseal cancellous bone leads to increased intraosseous pressure, accumulation of cyst fluid, and activation of local inflammatory mediators. Increased levels of prostaglandins, interleukins, and proteolytic enzymes in the cyst fluid may contribute to bone resorption and cyst enlargement. As skeletal growth progresses, the cyst may migrate from the metaphysis toward the diaphysis and may become less active over time. Plain radiography is typically the first-line imaging modality for diagnosis and follow-up, as UBCs usually demonstrate characteristic radiological features. In selected cases, computed tomography may be useful to evaluate cortical thinning, cyst wall integrity, and fracture risk, particularly in anatomically complex regions, whereas magnetic resonance imaging can help define lesion extent, fluid characteristics, aggressive features, and differential diagnosis from other cystic or lytic bone lesions.

It is often asymptomatic and discovered incidentally during imaging for other reasons but can also be diagnosed through spontaneous or trauma-related fractures [5,6]. Symptomatic lesions may occasionally lead to movement limitation and reduced participation in physical or athletic activities, particularly when pain, pathological fracture, cortical thinning, or mechanical instability is present. The most common symptom reported is pain due to a pathological fracture [7]. The most common complication, fracture, may lead to cyst ossification [8,9]. Differential diagnosis includes lesions such as aneurysmal bone cyst, fibrous dysplasia, non-ossifying fibroma, eosinophilic granuloma, enchondroma, and chondromyxoid fibroma [1,4,10]. Extension beyond the physis into the epiphysis can cause growth disturbances, leading to limb shortening or deformities [10]. The rarity of its occurrence in the adult population suggests that the condition may resolve on its own [1]. The goal of treatment is to prevent the risk of fracture and deformity development [1]. Various treatment approaches have been described, but there is no consensus. For conservative treatment, methods such as simple observation or activity restriction are recommended [11,12]. Percutaneous corticosteroid injection is a minimally invasive and effective method for treating UBC [13]. Elastic stable intramedullary nail, autologous bone marrow injection or combinations of these are also applied in the treatment of UBC [14]. Although lauromacrogol (polidocanol) has been more commonly described in the treatment of aneurysmal bone cysts, sclerosing agents have also been explored as minimally invasive intralesional treatment options for cystic bone lesions. In the present multicenter cohort, lauromacrogol was used in selected UBC cases as one of the percutaneous injection agents; therefore, it was included among the treatment modalities evaluated in this study [15]. Surgical treatment options include drainage, open curettage with grafting, and additionally, methods such as internal fixation, minimally invasive curettage with grafting, and cannulated screw placement for drainage have been described [10,16].

Although numerous studies have evaluated UBC, there is still no universally accepted treatment algorithm, and treatment selection remains influenced by lesion localization, fracture risk, radiological characteristics, and institutional experience. Moreover, many published studies are based on single-center cohorts or focus on a specific treatment modality. Therefore, multicenter real-world data comparing different treatment approaches are valuable for understanding current clinical practice and long-term outcomes. We hypothesized that individualized treatment selection based on patient- and lesion-related factors may result in favorable clinical and radiological outcomes across conservative, percutaneous, and surgical treatment modalities. The unique contribution of the present study is that it includes a large national pediatric cohort from seven referral centers, compares three commonly used treatment strategies, and evaluates both clinical and radiological outcomes with long-term follow-up. The aim of our study is to review and compare the diagnostic, follow-up, and treatment methods in cases from seven different clinics across the country.

## 2. Materials and Methods

This multicenter retrospective study included patients aged 0–20 years who were diagnosed with unicameral bone cyst between 2010 and 2023 followed in seven participating centers. The diagnosis, treatment, and follow-up processes of 180 UBC cases aged 0–20, followed in seven different clinics, were examined. Ethics committee approval is registered in the Ethics Committee of Bursa Uludağ University with decision number 2023-16/1 and approval date 1 August 2023.

Patients were eligible for inclusion if they had a radiological diagnosis compatible with UBC, available clinical and radiological records at diagnosis, documented treatment and follow-up data, and a minimum follow-up period of 12 months. A 12-month minimum follow-up period was selected to allow adequate evaluation of early clinical recovery, radiological regression, treatment failure, recurrence, and early complications after treatment. Patients were excluded if they had a final diagnosis other than UBC after radiological or histopathological review, insufficient clinical or radiological documentation, incomplete treatment records, missing follow-up data, or a follow-up period shorter than 12 months. Patients with secondary cystic bone lesions, malignant bone tumors, metabolic bone disease, or incomplete data preventing outcome assessment were also excluded.

The data collection form from the Pediatric Orthopedic Society Tumor Working Group—Aneurysmal Bone Cyst—Unicameral Bone Cyst was completed for all patients. The form collected demographic data, diagnosis date, the center where the diagnosis was made (either the following or another center), the initial point of contact (emergency department or outpatient clinic), symptoms at presentation, whether histopathological sampling was performed, and the center where the sample was taken. Diagnostic and follow-up imaging methods, as well as radiological and histopathological diagnoses, were reviewed. The diagnosis of UBC was primarily based on plain radiography, which was used as the standard first-line imaging modality in all patients. Additional imaging with MRI and/or CT was performed in selected cases to evaluate lesion extent, cortical integrity, fracture risk, aggressive radiological features, anatomical details, and differential diagnosis from other cystic or lytic bone lesions. The location and size of the lesion, epiphyseal involvement at diagnosis, and distance from the physis were noted. The presence of pathological fractures at diagnosis and during follow-up, as well as the healing time for fracture cases, were recorded. Radiological features at diagnosis were classified into latent, active, and aggressive stages according to the Enneking staging system. Histopathological examination was not performed in all patients. Biopsy and histopathological evaluation were available in 121 patients (67.2%), while the diagnosis in the remaining 59 patients (32.8%) was based on characteristic clinical and radiological findings compatible with a unicameral bone cyst. The presence of pathological fracture was recorded separately at initial diagnosis and during follow-up. A pathological fracture was present at diagnosis in 63 patients (35.0%), while 26 patients (14.9%) developed a pathological fracture during the follow-up period.

Treatment methods were divided into three groups: conservative, percutaneous injection, and open surgery. All participating centers were referral clinics with multidisciplinary musculoskeletal tumor boards. In these centers, patients were evaluated together with their radiological findings, and treatment decisions were made through a multidisciplinary decision-making process. A single standardized treatment algorithm was not applied uniformly across all centers, as UBCs may present with heterogeneous clinical and radiological features and may require individualized management. However, treatment selection was based on current accepted practice and guideline-based principles. In general, conservative management, percutaneous injection, or open surgery was selected according to lesion localization, symptoms, pathological fracture status, lesion size and radiological characteristics, proximity to the physis, mechanical loading of the affected bone, and the need for structural stabilization. Conservative management was generally preferred for asymptomatic or mildly symptomatic lesions with lower mechanical risk. Percutaneous injection was considered as a minimally invasive option in selected lesions requiring intervention without major structural instability. Open surgery was more commonly preferred for lesions with higher mechanical risk, lower-extremity involvement, large lesions, recurrent or insufficiently consolidated lesions, or cases requiring stabilization.

In general, conservative management was preferred for asymptomatic or mildly symptomatic lesions, lesions with lower mechanical risk, and lesions without major structural instability, particularly in the upper extremity. Percutaneous injection was considered as a minimally invasive option for symptomatic lesions or lesions requiring intervention but without a clear need for open stabilization. The choice between corticosteroid and lauromacrogol was based on multidisciplinary team preference, lesion characteristics, and institutional experience. Corticosteroid injection was generally used as a single-session intralesional treatment, whereas lauromacrogol was applied as a sclerosing agent according to repeated-injection protocols when this approach was preferred by the treating center. Open surgery was more commonly selected for lesions with higher mechanical risk, lower-extremity involvement, large lesions, recurrent or insufficiently consolidated lesions, pathological fractures requiring mechanical support, or cases in which structural stabilization was considered necessary. Within the surgical group, curettage with grafting was preferred when lesion debridement and cavity filling were considered sufficient, whereas internal fixation was added in cases with higher fracture risk, lower-extremity mechanical loading, large cortical defects, or instability requiring structural support.

Follow-up periods and investigations were recorded for conservative cases. In the percutaneous injection group, the type of agent, dose, and frequency were noted. For surgical cases, internal fixation was documented. If primary treatment failed, subsequent treatments were identified, and the reasons for treatment inadequacy were recorded. Cast, splint, or sling use for upper extremity lesions and non-weight-bearing periods for lower extremity lesions were also noted. Clinical recovery was assessed using Musculoskeletal Tumor Society (MSTS) scores, and radiological healing was classified into three stages according to the Capanna criteria. Radiological healing according to the Capanna criteria was assessed retrospectively using the available follow-up radiographs and clinical records at each participating center. The assessments were performed by the treating orthopedic oncology or pediatric orthopedic teams, with radiological evaluation when required. Because of the retrospective multicenter design, the images were not centrally re-evaluated by two independent blinded observers, and interobserver agreement was not calculated. Functional recovery was assessed using the Musculoskeletal Tumor Society (MSTS) scoring system before treatment, at the 3rd, 6th, 12th, and 24th months, and at the final follow-up. Follow-up durations and any early and late complications were recorded.

### Statistical Analysis

Statistical analysis was performed using IBM SPSS for Windows, Version 24 (Release 2016; IBM Corp., Armonk, NY, USA), with a 95% confidence level. *t*-tests, one-way ANOVA, repeated measures ANOVA, and chi-square tests were used. Comparisons of measurements between two groups were performed using *t*-tests, and comparisons among three groups were analyzed with one-way ANOVA. MSTS score changes were analyzed using repeated measures ANOVA. Relationships between categorical variables were examined using chi-square tests. Because of the retrospective multicenter design, no a priori power analysis was performed. All eligible patients who met the inclusion criteria during the study period and had adequate clinical, radiological, treatment, and follow-up data were included; therefore, the sample size was determined by the available cohort.

## 3. Results

A total of 180 patients from 7 centers were included in the study. The majority of patients were male (138 boys, 42 girls). The mean age at diagnosis was 10.27 ± 3.87 years (range: 2–20).

Half of the patients presented with pain. The majority of initial consultations were through outpatient clinics. Histopathological diagnosis was made in 2/3 of the cases, with biopsies mostly performed at the referring centers. Radiographs were used as the standard diagnostic method, and MRI was the most commonly requested additional imaging modality (77.2%). The radiological diagnosis had high accuracy (96.1%). The most commonly affected bones were the humerus (44.7%) and femur (30.7%). Although the humerus was most frequently involved, lower extremity involvement was not less frequent than upper extremity involvement. Bilateral involvement was seen in only one case. The most common location was the metadiaphyseal region. No lesions were primarily epiphyseal, but 8.3% extended into the epiphysis. Lesions were generally not far from the epiphysis, with an average distance of 28.7 mm. Pathological fractures were identified at diagnosis in 35% of cases, and 14.9% developed fractures during follow-up, meaning half of the cohort experienced a pathological fracture. The average healing time for fractures was 7.96 weeks. 62.2% of lesions were radiologically active at presentation.

Treatment methods included conservative management (19%), percutaneous injection (31.8%), and surgical treatment (49.2%). The average lesion volume was 47.65 cm^3^ (range: 0.11–291.60 cm^3^). In the upper extremity, more than half of the cases used casts or slings, while in the lower extremity, the average non-weight-bearing period was 5.67 ± 2.71 days (Table 1).

Radiographs were the standard monitoring method. MRI was the preferred follow-up modality in the conservative group (48.6%), but it was requested in only one surgical patient and not at all in the percutaneous injection group.

For treatment, percutaneous injections were more common in upper extremity lesions, while open surgery was more frequent for lower extremity lesions (Table 2).

### 3.1. Clinical Scores and Radiological Stage Changes

At the 2-year follow-up, clinical scores for the entire cohort nearly reached maximum values (Table 3). Radiologically, lesions showed stage regression. The average follow-up period was 81 months. Complications were rare, with skin issues being the most common early complication and limb shortening the most common late complication (Table 4).

**Figure 1:**
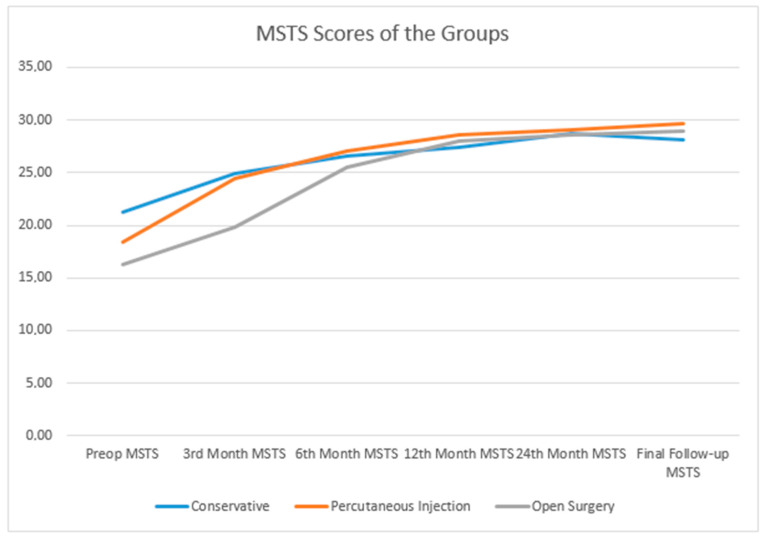
Clinical Score (MSTS) Follow-up of the Groups.

For clinical scores, conservative treatment patients had higher pre-treatment scores than other groups. By the third month, injection group scores matched those of the conservative group and were higher than the surgical group. At 6 months, scores balanced out, and at 2 years, scores were similar across groups. At the final follow-up, the percutaneous injection group had the highest scores. The greatest score improvement was seen in the surgical group (Table 5; Figure 1).

For protective procedures after treatment, the use of cast or sling in upper extremity lesions was independent of the chosen treatment, while in lower extremity lesions, longer non-weight-bearing periods were observed for surgical cases. According to Capanna criteria, although the conservative group was more advanced at baseline, stages balanced out post-treatment. Complication rates were similar across groups (Table 4). According to the Capanna criteria, pre-treatment radiological classification differed among treatment groups; however, post-treatment radiological classifications were more balanced, and no statistically significant difference was observed among conservative, percutaneous injection, and open surgery groups after treatment.

Initial radiological stage did not affect treatment choice. Interestingly, patients with more advanced radiological stages before treatment had higher clinical scores for 2 years. Post-treatment radiological staging, however, was unrelated to clinical scores (Table 6).

### 3.2. Percutaneous Injection Application and Outcomes

Percutaneous injection procedures were performed in the operating room under fluoroscopic guidance. After accessing the lesion, a sample was obtained for pathological examination, followed by contrast medium injection to determine the lesion volume and check for leakage. Subsequently, medication was administered into the lesion. Methylprednisolone was the most commonly preferred agent in the injection group, usually administered in a single dose (Table 7). Lauromacrogol was applied in repeated doses at 6-week intervals (Table 5).

In cases treated with lauromacrogol, clinical scores, initially lower than in corticosteroid-treated patients, caught up over time, with the final follow-up showing similar scores despite initially being behind. Score improvement was more significant in the lauromacrogol group. Pre-treatment radiological stages were more advanced in the corticosteroid group, but these stages balanced out after treatment. Lauromacrogol-treated patients had longer follow-up periods (Table 8).

### 3.3. Surgical Treatment Procedures and Outcomes

For patients undergoing open surgery (including those treated as secondary), curettage was the standard procedure, and nearly half received grafting, with some also receiving fixation. Additional treatment was rarely needed, with insufficient consolidation being the most common reason (Table 9). Autografts and/or allografts were used as grafts.

Comparing the most common surgical procedures (curettage with grafting vs. curettage with grafting and fixation), no significant differences were observed in clinical scores or radiological staging. Only at the 3-month mark did differences in MSTS scores appear, but they were not sustained over time (Table 10).

In the surgical treatment group, adding internal fixation was independent of the use of protective procedures in the upper extremity (cast or sling). In the lower extremity, however, the group without internal fixation showed longer periods of non-weight-bearing (Table 11).

### 3.4. Pathological Fractures: Localization and Effects

Pathological fractures were most commonly found in the metadiaphyseal region. The treatment modality and radiological stage were independent of lesion location (Table 12).

Lesion volume was not associated with pathological fractures at diagnosis or during follow-up. Although the surgical group had relatively larger lesions, lesion volume did not influence treatment choice (Table 13).

At diagnosis, pathological fractures did not affect treatment choice or clinical scores (Table 14).

## 4. Discussion

The main strength of this study is its comparison of three treatment approaches for UBC, offering detailed insights into clinical and radiological outcomes. By examining treatment progress, complications, and long-term results, it provides valuable information on the effectiveness of each method. Data from seven clinics across the country further contribute to the literature, making this study a key resource for improving clinical practice and understanding UBC treatment approaches.

UBC is more commonly seen in male patients during the first two decades of life [1]. The average age at diagnosis reported is 9 years [4]. Lesions are most commonly located in the humerus (50–75%) [1,17,18]. In this study, patients from the pediatric population were examined. The reported age at diagnosis was one year higher than in the literature (Table 1). The fact that the majority of cases (76.7%) were male and the most common locations were the humerus (44.7%) and femur (30.7%) supports the existing literature (Table 1).

UBC is considered typically a painless, asymptomatic lesion in the absence of a fracture, with symptoms reported in only 20% of cases [2]. In the presence of a pathological fracture, patients may present with pain, swelling, and deformity [10]. The most common symptom is pain associated with a pathological fracture [7]. In our cases, 35% had a pathological fracture at the time of first presentation, but in half of them, the symptom of “pain” was prominent (Table 1). The incidental detection of asymptomatic cases or the absence of hospital visits could explain this contrasting situation. However, we report that among those who presented to the hospital, symptoms, particularly pain, were commonly observed.

Radiological pre-diagnosis showed a high accuracy rate of 96.1%, with histopathological verification performed in two-thirds of cases (Table 1). While histopathological examination is rarely needed for UBC diagnosis [2], the high verification rate in our cohort supports the reliability of radiological diagnosis alone. However, for patients undergoing interventions, histopathological evaluation of samples taken during procedures may help confirm the diagnosis without causing harm and should be encouraged.

In radiological evaluation, plain radiography is the standard method for diagnosis [2]. CT can be used in areas where radiographic evaluation is more challenging, such as the spine and pelvis, to assess cyst wall thickness and evaluate fracture risk, providing additional value in differential diagnosis. MRI can be used as an additional investigation to evaluate the aggressive features of the lesion [2]. It also provides value in the differential diagnosis of other cystic lesions [19]. PET and scintigraphy are rarely used, primarily to exclude other conditions [1,2]. In our cases, MRI was most commonly requested in addition to radiography for diagnosis (Table 1). Radiography remains the standard imaging technique for follow-up, providing information about the lesion’s progression [20]. It appears that MRI was used for follow-up imaging only in the group under conservative observation.

Lesions are most commonly seen in the proximal humerus and femur. Diaphyseal lesions are more frequently observed in adults and are associated with the migration of metaphyseal lesions [1]. In our cases, lesions were most commonly localized in the metadiaphyseal region and were on average 28.7 mm away from the epiphysis (Table 1). Lesions within 20 mm of the physis have a higher risk of recurrence, and healing has been reported to be negatively affected in these cases [21]. However, it has also been suggested that this may be related to surgeons being less aggressive in their approach to protect the physis [10]. If it is accepted that lesions within 20 mm of the physis negatively affect healing, as reported in previous studies [21], the treatment of lesions, which are generally not far from the epiphysis as we observed, should involve patient-specific approaches with close monitoring and management.

Conservative monitoring or percutaneous injection is recommended for upper extremity lesions, while surgery is suggested for symptomatic lower extremity lesions [1,4,7]. The lower surgical preference for upper extremity lesions may be explained by the lack of mechanical threat due to their non-weight-bearing natüre [1]. In our cohort, non-surgical methods were preferred in 74.4% of upper extremity lesions, while surgery was preferred in 71% of lower extremity lesions (Table 2).

UBC treatment protocols are successful, leading to good clinical outcomes [1,2,4,22]. In our cases, the highest score improvement was observed in the open surgery group (Table 5, Figure 1), while the highest final follow-up score was in the percutaneous injection group. However, after 2 years, all procedures resulted in near-perfect clinical scores (Table 3). Differences in score changes could be balanced by personalizing treatment. However, personalization should not rely solely on radiological evaluation, pathology, or the presence of pathological fractures. In our cohort, radiological stage did not influence treatment choice, and paradoxically, patients with more advanced pre-treatment stages had higher clinical scores over the following 2 years. Post-treatment radiological staging was unrelated to clinical scores (Table 5). Pathological fractures at diagnosis did not affect treatment choice or clinical outcomes (Table 14). Therefore, personalized treatment should primarily be guided by lesion localization, clinical symptoms, fracture status, radiological and morphological characteristics, proximity to the physis, and mechanical risk. In the context of multidisciplinary musculoskeletal tumor boards, these factors can be integrated with current accepted practice to support individualized treatment planning.

The risk of pathological fractures, which can be as high as 90% at initial presentation in the literature [23], was observed to be 35% in our cohort. In follow-up, it was observed that half of the patients experienced a pathological fracture (Table 1).

The goal of treating UBC is to prevent pathological fractures and related deformities, while ensuring normal functional activity [4,18]. For this reason, various treatment methods have been defined, but no consensus exists on the optimal approach [4]. In our study, nearly half of the UBC patients unfortunately experienced pathological fractures. While 35% had a fracture at diagnosis, the incidence dropped to 14.9% in those who started treatment and follow-up without fractures. The study demonstrated that successful treatment was achieved in cases with fractures, regardless of the modality used.

The 2023 study by Strohn et al. [13] shows that, unlike in the past when surgery was almost the only treatment for UBCs, reports in the specialist literature of the last 40 years increasingly describe different, sometimes complex, treatment procedures that vary greatly in their level of invasiveness. These include steroid injections. In 2013, Flont et al. [24] argued that this method remains effective and may be particularly beneficial in early-stage lesions. In our study, we reaffirm that this method remains effective. Of the 41 patients who received methylprednisolone acetate, 40 required no additional treatment and were associated with good clinical outcomes. Another intralesional injection option, lauromacrogol (polidocanol), commonly used in the treatment of aneurysmal bone cysts, has also shown evidence of promoting cyst ossification. Strohm et al. [13] stated in their study that injection treatments of steroids and other effective agents are applied in the treatment of UBC, but it could not be clearly formulated due to the lack of available homogeneous group studies. Kumar et al. [25] reported a 92.3% rate of ossification with intralesional sclerotherapy in a study of 26 cases, while Puthoor et al. [26] demonstrated that sclerotherapy is comparable to curettage and grafting. In our study, we show that intralesional sclerotherapy can also be effective in the treatment of UBC, with near-complete clinical scores at the final follow-up in 13 cases (Table 8). Although some of these patients were at earlier radiological stages and had lower clinical scores, the results at 2 years were comparable to those treated with corticosteroids. We provide a breakdown of the selection and application methods for each injection agent (Table 6 and Table 7).

Bukva et al. [27] argued that surgical curettage and grafting led to better outcomes and a lower risk of recurrence compared to steroid injections. However, due to its low cost and ease of application, they recommended intralesional steroid injection as an initial treatment option. Similarly, in a study comparing minimally invasive methods, it was argued that percutaneous curettage showed higher success rates than intralesional injection [28]. In addition, Strohm et al. [13] reported in their study that excellent results were obtained with elastic intramedullary nailing applications in the treatment of UBCs and that additional studies were needed for valid recommendations. In our study, only one case showed recurrence among the surgically treated cases. Three cases required additional surgical intervention due to insufficient consolidation, and two cases due to fractures. In the percutaneous injection group, only one case required additional intervention due to insufficient consolidation. The findings indicate that percutaneous injection, even as a final treatment option, is also associated with good clinical outcomes.

In the preferred surgeries, curettage and grafting were most commonly performed, often with the addition of internal fixation (Table 9). No significant differences were observed between the preferred surgical methods and clinical scores, except at the postoperative 3-month mark. Additionally, no differences were found in the radiological staging (Table 10). However, in lower extremity lesions, internal fixation facilitated early mobilization (Table 11). It can be argued that the need for fixation in lesions of different localizations, sizes, and mechanical loads should be evaluated on a lesion-specific basis.

Radiological healing can be evaluated using the Capanna criteria [13,29]. It has been suggested that different Capanna stages may have an impact on clinical scores [30]. In our study, lesions that were mostly in stage 2 at the time of initial presentation showed a 75% transition to stage 1 after treatment (Table 4). However, contrary to the Capanna stage-clinical score relationship reported in the literature [30], our larger patient group did not show a significant difference in this regard (Table 5).

Recurrence and limb length discrepancy complications may occur after treatment [1,30]. In our patient group, complications were rare, with skin issues being the most common early complication, while limb length discrepancy was the most frequent late complication (Table 4).

In UBC’s the most effective treatment appears to be the association of curettage, bone graft, and elastic stable intramedullary nail [12]. Decompressing the cyst wall is more critical to increase the healing rate than the type of graft used for filling the cavity. Deventer et al. [7] suggested that, while upper extremity cysts can be managed conservatively without treatment if no fracture occurs, surgical treatment is more suitable for lower extremity cysts to promote early mobilization and prevent deformity. Our findings support this view, as conservative management in upper extremity lesions resulted in good clinical scores (Table 7). However, in contrast, surgical treatment of lower extremity lesions was associated with a significantly longer period of non-weight bearing compared to other treatments (Table 4). Nonetheless, the addition of internal fixation was linked to earlier mobilization in lower extremity lesions (Table 11).

UBC typically starts in the metaphysis and migrates towards the epiphysis, but can also be found in other locations [10]. In our study, pathological fractures were most common in lesions located in the “metadiaphysis” (Table 12). Location did not affect fracture development, radiological classification, or treatment choice during follow-up. Our data contributes to the literature by showing that lesions with pathological fractures are most commonly located in the “metadiaphysis.”

We observed that lesion volume did not contribute to fracture development. Although not statistically significant, there was a trend suggesting that as the lesion size increased, more aggressive treatments were preferred (Table 13).

Unfortunately, pathological fractures developed in half of the patients; however, they did not have a negative impact on clinical scores, which is being reported for the first time (Table 14). Pathological fracture is one of the most clinically relevant presentations of UBC. In our cohort, 35.0% of patients had a pathological fracture at diagnosis, and additional fractures occurred during follow-up. However, when patients were divided according to the presence or absence of pathological fracture at presentation, no significant difference was observed in treatment modality, follow-up MSTS scores, final MSTS scores, or MSTS improvement. This finding suggests that pathological fracture at diagnosis does not necessarily compromise long-term functional recovery when patients are managed appropriately. This is clinically important because UBCs are often diagnosed after fracture-related symptoms. Donaldson et al. emphasized that evidence remains limited and that comparative outcome data are needed, while current clinical summaries also recognize pathological fracture as the main symptomatic presentation of UBC [6]. Our data contribute to this discussion by showing, in a multicenter cohort, that fracture at presentation did not adversely affect long-term functional outcome.

A study found that pathological fractures healed in 8 weeks with curettage, grafting, and intramedullary nailing and the final MSTS score was 28.5, with 94% excellent outcomes [22]. In our study, healing took 7.96 weeks (Table 1), with similar healing times and MSTS scores, consistent with the literature.

One limitation of this study is related to the radiological outcome assessment. Radiological healing according to the Capanna criteria was evaluated retrospectively using the available follow-up radiographs and medical records from each participating center. Although the assessments were performed by experienced orthopedic oncology or pediatric orthopedic teams, the images were not centrally re-evaluated by two independent blinded observers, and interobserver agreement was not assessed. This may have introduced observer-related variability in radiological classification. Another limitation of this study is its retrospective, non-randomized design. Treatment allocation was not randomized and may have been influenced by lesion characteristics, anatomical localization, mechanical risk, multidisciplinary team preference, and institutional practice. Therefore, direct comparisons among conservative management, percutaneous injection, and open surgery should be interpreted with caution and should not be considered as definitive evidence of the superiority of one treatment modality over another. Nevertheless, the multicenter design, long follow-up period, and inclusion of cases managed in referral centers with multidisciplinary musculoskeletal tumor boards provide valuable real-world data on individualized treatment strategies for UBC. In addition, no a priori power analysis was performed because of the retrospective design, and the sample size was determined by the number of eligible patients available during the study period. However, the multicenter nature of the study, the inclusion of 180 patients, and the long follow-up period provide valuable real-world data on the diagnosis, treatment, and follow-up of UBC.

## 5. Conclusions

As a conclusion, this study evaluates the effectiveness of different approaches in the diagnosis, follow-up, and treatment of UBC. Conservative treatment, percutaneous injection, and surgical methods were compared, showing significant improvements in clinical scores and radiological stages. The surgical group had the highest score increase, while both corticosteroid and sclerosing injections showed positive results, with sclerosing injections demonstrating better long-term clinical outcomes.

Radiological stage did not influence treatment choice, and pathological fractures did not affect clinical outcomes. In conclusion, treatment should be personalized based on the patient’s characteristics and lesion type. All three approaches were effective and reliable.

This study provides valuable insights for developing standardized treatment protocols for UBC and represents the largest cohort in the country, offering a detailed overview of the current patient population.

## Figures and Tables

**Table 1:** Clinical and Radiological Features.

		*n* (%)
Symptoms	Pain	92 (51.1%)
	Pathological Fracture	63 (35%)
	Loss of Function	19 (10.6%)
	Swelling	22 (12.2%)
	Deformity	6 (3.3%)
Initial Consultation	Outpatient Clinic	114 (63.3%)
	Emergency	66 (36.7%)
Was a Biopsy Performed?	Yes	121 (67.2%)
	No	59 (32.8%)
Biopsy Center	Same Center	114 (94.2%)
	External Center	7 (5.8%)
Imaging Methods	X-ray	180 (100%)
	MRI	139 (77.2%)
	CT (Computed Tomography)	27 (15%)
	Bone Scintigraphy	16 (8.9%)
	PET (Positron Emission Tomography)	5 (2.8%)
Radiological Diagnosis	Unicamaral Bone Cyst (UBC)	172 (96.1%)
	Aneurysmal Bone Cyst (ABC)	6 (3.4%)
	Fibrous Dysplasia	1 (0.6%)
Affected Bone	Humerus	80 (44.7%)
	Ulna	2 (1.1%)
	Radius	3 (1.7%)
	Pelvis	1 (0.6%)
	Femur	55 (30.7%)
	Tibia	15 (8.4%)
	Fibula	11 (6.1%)
	Calcaneus	11 (6.1%)
	Finger Bones	1 (0.6%)
Upper/Lower Extremity	Upper	86 (48%)
	Lower	93 (52%)
Side	Right	101 (56.4%)
	Left	77 (43%)
	Bilateral	1 (0.6%)
Location	Epiphysis	0 (0%)
	Diaphysis	62 (35%)
	Metaphysis	35 (19.8%)
	Metadiaphysis	80 (45.2%)
Epiphyseal Involvement	Yes	15 (8.3%)
	No	165 (91.7%)
Distance from Physis (mm) (Mean ± SD)		28.70
Pathological Fracture at Diagnosis?	Yes	63 (35%)
	No	117 (65%)
Pathological Fracture During Follow-up?	Yes	26 (14.9%)
	No	148 (85.1%)
Healing Time for Pathological Fractures (weeks)		7.96 ± 3.61 (4–16)
Radiological Staging at Diagnosis (Enneking)	Latent	43 (23.9%)
	Active	112 (62.2%)
	Aggressive	25 (13.9%)
Treatment Modality	Conservative	35 (19.4%)
	Percutaneous Injection	57 (31.7%)
	Open Surgery	88 (48.9%)
Lesion Size at Diagnosis (mm)		(Mean ± SD)
	Coronal	51.51 ± 31.2
	Sagittal	29.98 ± 17.35
	Axial	24.96 ± 11.89
	Total Volume (cm^3^)	47.65 ± 49.18 (0.11–291.60)
Upper Extremity Cast/Sling	Yes	75 (56%)
	No	59 (44%)
Lower Extremity Non-weight-bearing Period (Weeks) (Mean ± SD)		5.67 ± 2.71

**Table 2:** Selected Treatment Modalities in Extremities.

		Upper/Lower Extremity		*p*
		Upper	Lower	
		*n* (%)	*n* (%)	
Treatment Modality	Conservative	22 (25.6%)	13 (14%)	0.000
	Percutaneous Injection	42 (48.8%)	14 (15%)	
	Open Surgery	22 (25.6%)	66 (71%)	

Chi-square Test performed.

**Table 3:** Clinical Score (MSTS) Follow-up of the Groups.

	Treatment Modality			*p* ^a^	Multiple Comparisons
	Conservative	Percutaneous Injection	Open Surgery		
	Mean ± SD	Mean ± SD	Mean ± SD		
Preop MSTS	21.26 ± 8.97	18.44 ± 5.92	16.32 ± 7.25	0.005	1 > 3
3rd Month MSTS	24.96 ± 5.02	24.46 ± 4.94	19.84 ± 5.66	0.000	1 > 3; 2 > 3
6th Month MSTS	26.56 ± 4.16	27.09 ± 3.58	25.47 ± 3.75	0.105	
12th Month MSTS	27.36 ± 4.88	28.61 ± 2.33	27.97 ± 2.68	0.313	
24th Month MSTS	28.67 ± 3.04	29.1 ± 1.45	28.59 ± 3.07	0.658	
Final Follow-up MSTS	28.1 ± 3.1	29.71 ± 0.6	28.98 ± 1.63	0.000	1 < 2; 2 > 3
Δ(MSTS) (Final Follow-up—Preop)	9.9 ± 10.53	11.67 ± 6.34	14.13 ± 8.62	0.039	1 < 3

^a^ Inter-group: One-way ANOVA test.

**Table 4:** Comparison of Protective Procedures, Radiological Stages, and Complications of Treatment Modalities.

		Treatment Modality			*p*	Multiple Comparisons
		Conservative	Percutaneous Injection	Open Surgery		
		*n* (%)	*n* (%)	*n* (%)		
Cast/Sling for Upper Extremity ^b^	Evet	16 (57.15%)	23 (57.5%)	34 (53.1%)	0.887	
	Hayır	12 (42.85%)	17 (42.50%)	30 (46.9%)		
Non-weight-bearing Period for Lower Extremity (Weeks) (Mean ± SD) ^a^		3.9 ± 3.84	3.27 ± 2.8	6.44 ± 2.01	0.000	1 < 3; 2 < 3
Pre-treatment Radiological Classification, Capanna ^b^	1	9 (26.5%)	25 (43.85%)	45 (51.7%)	0.041	
	2	24 (70.5%)	32 (56.15%)	42 (48.3%)		
	3	1 (3%)				
Post-treatment Radiological Classification, Capanna ^b^	1	25 (71.4%)	46 (80.7%)	62 (72.9%)	0.259	
	2	10 (28.6%)	11 (19.3%)	23 (27.1%)		
Early Complications ^b^	None	31 (96.9%)	54 (98.2%)	84 (96.6%)	0.238	
	Skin Issues	0 (0%)	1 (1.8%)	2 (2.3%)		
	Infection	1 (3.1%)	0 (0%)	0 (0%)		
	Pathological Fracture	0 (0%)	0 (0%)	1 (1.1%)		
Late Complications ^b^	None	30 (93.75%)	49 (89.1%)	74 (88.1%)	0.089	
	Limb Shortening	0 (0%)	0 (0%)	7 (8.4%)		
	Deformity	0 (0%)	3 (5.5%)	1 (1.1%)		
	Pathological Fracture	0 (0%)	1 (1.8%)	1 (1.1%)		
	Skin Issues	2 (6.25%)	2 (3.6%)	0 (0%)		
	Recurrence	0 (0%)	0 (0%)	1 (1.1%)		

^a^ = ANOVA test, ^b^ = Chi-square test.

**Table 5:** Application Methods of Injection Agents.

		Which Agent			*p*
		Prednisolone	Methylprednisolone	Lauromacrogol	
		*n* (%)	*n* (%)	*n* (%)	
What Dose (mg) (Mean ± SD) ^a^		142.5 ± 74.11	89.3 ± 25.95		0.247
How Many Injections ^b^	1	3 (60%)	36 (83.7%)	2 (16.7%)	0.000
	2	0 (0%)	6 (14%)	6 (50%)	
	3	2 (40%)	0 (0%)	3 (25%)	
	4	0 (0%)	1 (2.3%)	1 (8.3%)	
How Often (weeks) (Mean ± SD) ^c^		1 ± 0	16.29 ± 16.05	6.56 ± 2.19	0.072

^a^ = *t* test, ^b^ = Chi-square test, ^c^ = ANOVA test.

**Table 6:** Relationship Between Radiological Stage and Clinical Score.

		Post-Treatment Radiological Classification, Capanna		*p*
		1	2	
		*n* (%)	*n* (%)	
Treatment Modality ^b^	Conservative	24 (18%)	11 (25.6%)	0.679
	Percutaneous Injection	44 (33%)	12 (27.9%)	
	Open Surgery	65 (49%)	20 (46.5%)	
Preop MSTS (Mean ± SD) ^a^		17.72 ± 7.47	18.67 ± 7.14	0.478
3rd Month (Mean ± SD) ^a^		22.6 ± 5.69	23.71 ± 5.58	0.358
6th Month (Mean ± SD) ^a^		26.35 ± 3.74	26.52 ± 4.02	0.844
12th Month (Mean ± SD) ^a^		28.31 ± 2.69	27.95 ± 3.35	0.603
24th Month (Mean ± SD) ^a^		28.95 ± 2.26	28.53 ± 2.59	0.533
Final Follow-up (Mean ± SD) ^a^		29.18 ± 1.77	28.63 ± 1.99	0.101
Δ(MSTS) (Final Follow-up—Preop) (Mean ± SD) ^a^		12.75 ± 8.42	11.68 ± 8.14	0.489

^a^ = *t* test, ^b^ = Chi-square test.

**Table 7:** Applications in the Injection Group.

		*n* (%)
Which Agent	Prednisolone	5 (8.3%)
	Methylprednisolone	43 (71.7%)
	Lauromacrogol	12 (20%)
Which Agent	Corticosteroid	48 (78.7%)
	Lauromacrogol	13 (21.3%)
What Dose (mg)		93.83 ± 34.61
How Many Injections	1	41 (67.2%)
	2	12 (19.7%)
	3	6 (9.8%)
	4	2 (3.3%)
How Often (weeks) (Mean ± SD)		9 ± 10.82

**Table 8:** Clinical and Radiological Comparisons of Injection Agents.

		Which Agent		*p*
		Corticosteroid	Lauromacrogol	
		*n* (%)	*n* (%)	
Preop MSTS (Mean ± SD) ^a^		19.93 ± 5.6	12.69 ± 2.63	0.000
3rd Month (Mean ± SD) ^a^		26.63 ± 2.67	16.38 ± 2.1	0.000
6th Month (Mean ± SD) ^a^		28.7 ± 1.87	21.15 ± 1.52	0.000
12th Month (Mean ± SD) ^a^		29.57 ± 0.93	25.31 ± 2.69	0.000
24th Month (Mean ± SD) ^a^		29.82 ± 0.46	27 ± 1.18	0.000
Final Follow-up (Mean ± SD) ^a^		29.74 ± 0.57	29.54 ± 0.78	0.309
Δ(MSTS) (Final Follow-up—Preop) (Mean ± SD) ^a^		10.28 ± 6.32	16.85 ± 2.61	0.000
Pre-treatment Radiological Classification, Capanna ^b^	1	11 (22.9%)	10 (76.9%)	0.001
	2	37 (77.1%)	3 (23.1%)	
Post-treatment Radiological Classification, Capanna ^b^	1	36 (76.6%)	11 (84.6%)	0.713
	2	11 (23.4%)	2 (15.4%)	
Follow-up Duration (Months) (Mean ± SD) ^a^		44.36 ± 32.06	119.85 ± 53.18	0.000

^a^ = *t* test, ^b^ = Chi-square test.

**Table 9:** Methods in the Open Surgery Group and Reasons for Additional Treatment in Cases.

		*n* (%)
Open Surgery	Curettage	2 (2.2%)
	Curettage + Grafting	39 (42.9%)
	Curettage + Grafting + Fixation	48 (52.7%)
	Curettage + Fixation	0 (0%)
	Curettage + Cementation	2 (2.2%)
Was internal fixation performed?	No	43 (47.3%)
	Yes	48 (52.7%)
Additional Treatment	None	71 (86.6%)
	Grafting	7 (8.5%)
	Fixation	2 (2.4%)
	Grafting and Fixation	2 (2.4%)
Reason for Additional Treatment	Insufficient consolidation	6 (54.5%)
	Progression	1 (9.1%)
	Recurrence	1 (9.1%)
	Fracture	2 (18.2%)
	Non-union	1 (9.1%)

**Table 10:** Comparison of Clinical Scores and Radiological Stages of Two Different Surgical Procedures.

		Open Surgery		*p*
		Curettage + Grafting	Curettage + Grafting + Fixation	
		*n* (%)	*n* (%)	
Preop MSTS (Mean ± SD) ^a^		16.53 ± 6.53	15.87 ± 8.2	0.700
3rd Month (Mean ± SD) ^a^		21.68 ± 4.99	18.1 ± 6.37	0.036
6th Month (Mean ± SD) ^a^		25.93 ± 3.66	25.37 ± 3.88	0.632
12th Month (Mean ± SD) ^a^		28.33 ± 2.37	27.88 ± 2.96	0.605
24th Month (Mean ± SD) ^a^		28.13 ± 3.74	29.27 ± 1.62	0.349
Final Follow-up (Mean ± SD) ^a^		28.77 ± 1.55	29.25 ± 1.58	0.167
Δ(MSTS) (Final Follow-up—Preop) (Mean ± SD) ^a^		14.75 ± 8.63	14.18 ± 8.99	0.766
Pre-treatment Radiological Classification, Capanna ^b^	1	24 (52.2%)	23 (56.1%)	0.880
	2	22 (47.8%)	18 (43.9%)	
Post-treatment Radiological Classification, Capanna ^b^	1	36 (78.3%)	29 (74.4%)	0.868
	2	10 (21.7%)	10 (25.6%)	
Follow-up Duration (Months) (Mean ± SD) ^a^		107.17 ± 111.37	106.94 ± 93.52	0.992

^a^ = *t* test, ^b^ = Chi-square Test.

**Table 11:** Relationship Between Internal Fixation and Protective Procedures.

		Was Internal Fixation Performed?		*p*
		No	Yes	
		*n* (%)	*n* (%)	
Cast/Sling for Upper Extremity ^b^	Yes	15 (57.7%)	22 (68.75%)	0.384
	No	11 (42.3%)	10 (31.25%)	
Non-weight-bearing Duration for Lower Extremity (Weeks) (Mean ± SD) ^a^		7.22 ± 1.51	5.42 ± 1.86	0.003

^a^ = *t* test, ^b^ = Chi-square Test.

**Table 12:** Data According to Localization.

		Localization			*p*
		Diaphysis	Metaphysis	Metadiaphysis	
		*n* (%)	*n* (%)	*n* (%)	
Is there a pathological fracture at diagnosis?	Yes	15 (24.2%)	11 (31.4%)	37 (46.3%)	0.021
	No	47 (75.8%)	24 (68.6%)	43 (53.8%)	
Did a pathological fracture occur during follow-up?	Yes	7 (12.3%)	4 (11.4%)	15 (18.8%)	0.460
	No	50 (87.7%)	31 (88.6%)	65 (81.3%)	
Treatment Modality	Conservative	16 (25.8%)	5 (14.3%)	13 (16.25%)	0.486
	Percutaneous Injection	19 (30.6%)	10 (28.6%)	28 (35%)	
	Open Surgery	27 (43.5%)	20 (57.1%)	39 (48.75%)	
Pre-treatment Radiological Classification, Capanna	1	32 (53.3%)	12 (34.3%)	34 (42.5%)	0.174
	2	28 (46.7%)	23 (65.7%)	46 (57.5%)	
Post-treatment Radiological Classification, Capanna	1	46 (76.7%)	28 (82.4%)	58 (72.5%)	0.523
	2	14 (23.3%)	6 (17.6%)	22 (27.5%)	

Chi-square Test performed.

**Table 13:** Relationship Between Lesion Volume and Pathological Fracture, and Treatment Selection.

		Total Volume	*p*
		Mean ± SD	
Is there a pathological fracture at diagnosis? ^a^	Yes	52.81 ± 51.23	0.353
	No	45.27 ± 48.25	
Did a pathological fracture occur during follow-up? ^a^	Yes	58.65 ± 51.8	0.251
	No	46.03 ± 48.2	
Treatment Modality ^b^	Conservative	31.43 ± 48.67	0.052
	Percutaneous Injection	46.11 ± 45.5	
	Open Surgery	55.64 ± 50.68	

^a^ = *t* test, ^b^ = ANOVA test.

**Table 14:** Relationship Between Pathological Fracture and Clinical Score.

		Is There a Pathological Fracture at Diagnosis?		*p*
		Yes	No	
		*n* (%)	*n* (%)	
Treatment Modality ^b^	Conservative	9 (14.3%)	26 (22.2%)	0.103
	Percutaneous Injection	26 (41.3%)	31 (26.5%)	
	Open Surgery	28 (44.4%)	60 (51.3%)	
Preop MSTS (Mean ± SD) ^a^		17.14 ± 7.6	18.48 ± 7.27	0.268
3rd Month (Mean ± SD) ^a^		22.44 ± 5.78	23.1 ± 5.65	0.535
6th Month (Mean ± SD) ^a^		26.61 ± 3.59	26.32 ± 3.89	0.699
12th Month (Mean ± SD) ^a^		28.5 ± 2.64	28.09 ± 2.93	0.479
24th Month (Mean ± SD) ^a^		29.23 ± 1.79	28.64 ± 2.58	0.274
Final Follow-up (Mean ± SD) ^a^		28.98 ± 2.12	29.12 ± 1.64	0.643
Δ(MSTS) (Final Follow-up—Preop) (Mean ± SD) ^a^		12.69 ± 7.91	12.49 ± 8.75	0.882

^a^ = *t* test, ^b^ = Chi-square Test.

## Data Availability

The data presented in this study are available on request from the corresponding author.

## Abbreviation

The following abbreviation is used in this manuscript:

UBC	Unicameral bone cyst
